# Patterned Reed–Muller Sequences with Outer A-Channel Codes and Projective Decoding for Slot-Controlled Unsourced Random Access

**DOI:** 10.3390/s23115239

**Published:** 2023-05-31

**Authors:** Wenjiao Xie, Huisheng Zhang

**Affiliations:** School of Electronics and Information, Northwestern Polytechnical University, Xi’an 710129, China; zhanghuisheng@nwpu.edu.cn

**Keywords:** block fading channels, machine-to-machine communications, unsourced random access, geometry theory, projective decoder, error correcting codes, complex Reed–Muller codes

## Abstract

We propose a novel slot-pattern-control based coded compressed sensing for unsourced random access with an outer A-channel code capable of correcting *t* errors. Specifically, an RM extension code called patterned Reed–Muller (PRM) code is proposed. We demonstrate the high spectral efficiency due to its enormous sequence space and prove the geometry property in the complex domain that enhances the reliability and efficiency of detection. Accordingly, a projective decoder based on its geometry theorem is also proposed. Next, the “patterned” property of the PRM code, which partitions the binary vector space into several subspaces, is further extended as the primary principle for designing a slot control criterion that reduces the number of simultaneous transmissions in each slot. The factors affecting the chance of sequence collisions are identified. Finally, the proposed scheme is implemented in two practical outer A-channel codes: (i) the *t*-tree code and (ii) the Reed–Solomon code with Guruswami–Sudan list decoding, and the optimal setups are determined to minimize SNR by optimizing the inner and outer codes jointly. In comparison with the existing counterpart, our simulation results confirm that the proposed scheme compares favorably with benchmark schemes regarding the energy-per-bit requirement to meet a target error probability as well as the number of accommodated active users in the system.

## 1. Introduction

The continuous evolution of massive machine type communication (mMTC+) [1] will still be one of main use cases of sixth-generation (6G) wireless networks [2,3]. In this context, the battery-limited terminals sporadically connected to the network grow exponentially and they are expected to send short information packets for low transmission latency [4,5].

The most promising way is to address grant-free transmission, i.e., the device transmits the packet without requiring coordination among users [6,7,8]. The “Sourced random access (SRA)” case refers to assigning separate dictionaries to individual users, i.e., employing different encoders for users [9,10]. Due to a large number of users, the SRA mechanism will result in exorbitant complexity as the AP does not know which decoder to utilize and is forced to try all possibilities. Therefore, employing the same coding protocol for all users is the most promising approach. Such schemes are known as “Unsourced random access (URA)” [11]. By using this mechanism, AP is not required to recognize active users’ identities and all users are allowed to share the same codebook, avoiding complex identity assignment and authentication processes among a large number of users. Additionally, the access and transmission performance of the overall system are analyzed based on the per-user probability of error (PUPE) for all active users.

Since then, several papers have been published demonstrating fundamental limits for various massive access channel models and configurations (see, e.g., [12,13,14]). Many transmission schemes have been proposed to achieve performance as close to these fundamental limits as possible [15,16,17].

**The Background for Unsourced Random Access with Outer A-Channel Codes:** The URA benefited greatly from the outer A-channel codes [18,19], e.g., the coded compressed sensing is a divide-and-conquer scheme that uses random inner codes of capacity $2^{G}$ concatenated with an outer *G*-ary A-channel code, in which a tree code is a type of A-channel code designed for this purpose. This flexible structure made it easy to adapt to new channel models. Several subsequent studies on URA (e.g., [16,20,21,22,23,24]) made use of an outer A-channel code.

The field of study [19] for a tree code as the outer A-channel code is extended in paper [25], which leverages the approximate message passing (AMP) strategy in conjunction with sparse regression codes. The coded compressed sensing (CCS) strategy is further improved in [16], where the inner AMP decoder and the outer tree decoder are authorized to exchange soft information via a shared message-passing protocol. By using the same sensing matrix as in [19], the authors reduce complexity in [26]. The paper [27] proposes a coded demixing strategy to facilitate the joint detection of high dimensional sparse signals concerning different bases.

**The Background for Reed–Muller Sequence:** The second-order Reed–Muller-related codebook construction schemes have been widely utilized in compressed sensing [28,29,30,31]. For instance, in [28], the complexity of the chirp reconstruction is dependent on the number of measurements rather than the signal dimension since RM sequences are utilized to build deterministic measurement matrices. In [29], the idea of using a measurement matrix whose columns are codewords of a linear code is proposed. In addition, a quadratic reconstruction algorithm that takes advantage of the multivariable quadratic functions is further employed. According to [31], full-duplex compressed neighbor discovery is supported by on–off RM signatures. Additionally, RM sequences are regarded as excellent options for massive access in mMTC due to their large codebook capacity and ability to be used in low-complexity random access [32]. The non-orthogonal RM sequences are used as signatures for active device detection [33] which is the grant-based access scheme that suffers from low access efficiency and high signaling overhead in mMTC. As described in [34], an incremental massive random access scheme is proposed. Furthermore, RM sequences leveraged as the inner codes for the URA scheme are proposed in [35,36] and the tree coding is used for stitching messages in packetized and slotted transmissions.

In this paper, based on the “patterned” property of the proposed patterned Reed–Muller (PRM) sequences, we design a slot-pattern-control (SPC) criterion that corresponds one-to-one to an information segment to construct the slot occupation guideline for each active user. We then partition the users’ messages into several sub-blocks and add the same SPC segment in each block as a prefix to make up the input signal of the inner encoder. On this basis, each sequence related a single codeword from a proposed common codebook called patterned Reed–Muller codes. For recovering the PRM sequences received at the receiver, an algorithm exploiting the geometry of PRM sequences is proposed. Moreover, the outer tree code is replaced with an error-correcting code and the codewords with a distance at most *t* from the channel output are recovered by outer codes.

### 1.1. Main Contributions of the Paper

To summarize, our contributions are listed as follows:

- This paper designs a common codebook that optimizes URA energy efficiency performance by embedding zero bits in the second-order Reed–Muller sequences in accordance with a specific principle, which is the simplified version proposed by Pllaha et al. (2022) [37,38,39], and we denote it as a patterned Reed–Muller (PRM) code.
- Instead of the available common codebook that uses Reed–Muller (RM) properties in the binary domain, we explore its exclusive natures in the complex domain. In detail, we prove the algebraic and geometric properties of the second-order Reed–Muller sequence in the complex field, and related theories for PRM codes are also proven.
- A projective decoder is proposed for the PRM sequence and the fundamental theory for proposing such precise detection algorithm stems from its geometry property.
- The dependencies enlightened by the patterned property of PRM sequences prescribe how the information messages are mapped to the elements in a pool of slot-pattern controls (SPCs). The information message guides a single user to select the corresponding SPC from the pool and users randomly select SPCs as their transmission criteria to reduce collision chance.
- The factors affecting the reliability of the PRM detection are discussed. As a result of our simulations, we conclude that the proposed slot-pattern-control PRM-based scheme offers significant advantages in terms of error probability.
- Instead of the outer tree code proposed by Amalladinne et al. (2020) [19], we couple the proposed slot-pattern-control PRM-based scheme with two practical list recoverable codes. The first is a modification of the tree code called *t*-tree code and the second is based on the Reed–Solomon codes and Guruswami–Sudan list decoding algorithm. The optimal setups are determined to minimize SNR by optimizing the inner and outer codes jointly. In regimes of practical interest, the proposed scheme compares favorably with benchmark schemes regarding the energy-per-bit requirement to meet a target error probability as well as the number of accommodated active users in the system.

In Section 2, we introduce the system model. Several theorems of RM for the complex field are proved in Section 3. In Section 4, we construct the proposed PRM sequence and design a single-user decoding algorithm based on a proven theorem. Section 5 provides a high-level description of the transmitter architecture, decoding procedures and a proposed slot-pattern-control pool design, followed by Section 6 on the factors influencing the PRM detection ability. The performance of the proposed transmission schemes is assessed via numerical simulations in Section 7. Section 8 concludes the paper.

### 1.2. Notation

This paper uses lowercase letters to represent scalars. Column vectors and matrices are denoted by boldface letters in lower case and upper case, respectively. We represent $F_{2}^{m}$ as the binary field. Sym(m;2) denotes the group of binary m×m symmetric matrices. We will denote matrices (resp., vectors) with upper case (resp., lower case) bold letters. $A^{T}$ will denote the transpose and $A^{-T}$ will denote the inverse transposed span(A) column space and the row space of A. Since all our vectors are columns, we will typically deal with column spaces. $I_{m}$ will denote the m×m matrix (complex or binary). U(N) denotes the set of unitary N×N complex matrices and *H* will denote the conjugate transpose of a matrix. The length *N* column vectors of all zeros and all ones are denoted as $0_{N}$ and $1_{N}$, respectively. We use $A={\left[a_{i}\right]}_{i=1}^{I}$ to represent the I-element vector made up of the elements $a_{i}$, where i=1,⋯,I. The result of appending the column vector $B_{1}\in C^{T_{1}\times 1}$ to $B_{2}\in C^{T_{2}\times 1}$ has the form of $B=\left[B_{1};B_{2}\right]\in C^{(T_{1}+T_{2})\times 1}$. $0^{N}$ is an all-zero column vector of length *N*. $0_{\Upsilon \times \Upsilon }$ denotes a full-zero matrix of size Υ×Υ. We denote by $\mathrm{CN}\left(0,I_{N}\right)$ a complex standard normal random vector. The symbol $\left|\cdot \right|$ is the cardinality of a set. O(·) represents the complexity order. E[·] denotes the expectation operator and  D[·] denotes the variance operator. Pr[e] means a probability of the event *e*. The vector $v_{n-1}$ is the decimal number (n−1), while conversely we have that $\mathrm{decimal}(v_{n-1})=n$, i.e., decimal(·) converts the binary vector to the corresponding decimal number and then increases the result by one.

## 2. System Model

In this paper, let us reuse the system proposed in [19,36]. Users are always silent in the absence of information to transmit. There are *K* potential users in the system and $K_{a}$ components are active, which typically means $K_{a}$ is far less than *K* in the mMTC scenario due to the infrequent activity of users. $K_{a}$ users transmit codewords drawn at random from a common codebook containing entries derived from a *G*-ary alphabet $\left[G\right]=\left\{1,\cdots ,G\right\}$ over *T* channel uses. The relationship between inputs and outputs of a channel over *H* slots is expressed as

(1) $$Y=\overset{K_{a}}{\underset{k=1}{⋃}}\left[C_{k,1},\cdots ,C_{k,H}\right],$$

where $C_{k,h}\in \left[G\right]$ represents the inner codeword sent by user *k* in the *m*-th message segment, 1≤h≤H, and the length of the codeword is $N=2^{m}$.

The expectation is that each active user will send *B* bits of information to the receiver utilizing *T* channel uses. Specifically, the set $\left\{W_{1},W_{2},\cdots ,W_{K_{a}}\right\}$ for *B*-bit messages, which contain all messages sent by $K_{a}$ users, are encoded into *H*-length *G*-ary A-channel codes, where $W_{k}\in \left[N\right]$, $\left[N\right]=\left\{1,\cdots ,N\right\}$ and $N=2^{B}$. We then define the URA code for the A-channel as:

$$\mathrm{encoder}\;E_{n}:\left[N\right]\mapsto {\left[G\right]}^{H};$$

decoderD^:⋃k=1K[G]k,⋯,⋃k=1K[G]k︷NTtimes↦[N]K,

where *K* is the number of simultaneous appearances in each block, $K<K_{a}$. We require ${∥E_{n}\left(W\right)∥}_{2}^{2}\leq HNP$, which means the power limitation. In this article, a proposed slot-pattern-control technique is used to carry out the packetized and slotted transmission; hence, the *H*-length outer codes are distributed throughout $N_{T}$ slots and $T=N\cdot N_{T}$.

At the receiver, after detecting codewords in each slot, the A-channel decoder is used to couple the divided information chunks. Here, a quasi-static Rayleigh fading channel is considered, in which the channel remains constant inside a single slot but fluctuates independently between slots. We assess performance from the perspective of messages and use the miss detection rate (MDR) and the false-alarm rate (FAR) as the primary indicators, which are given as

(2) $$P_{e}≜\frac{1}{K_{a}}\sum_{k\in K_{a}}\mathrm{Pr}\left[W_{k}\notin \hat{D}\left(Y\right)\right],$$

and

(3) $$P_{f}≜\mathrm{Pr}\left[\hat{D}\left(Y\right)\backslash \left\{W_{k}|k\in K_{a}\right\}\neq \emptyset \right],$$

respectively, where $K_{a}=\left\{1,\cdots ,K_{a}\right\}$. Energy efficiency is of critical importance for the mMTC scenario; our goal is to minimize the energy per bit ($E_{b}/N_{0}=HNP/B$) spent by each user.

## 3. Reed–Muller Sequences

The existing unsourced random access systems using Reed–Muller sequences primarily investigate the RM features in the binary domain [35]. In reality, the complex RM sequences are unit norm vectors on $C^{N}$, which exhibit subtle algebraic and geometric properties. In this section, we present a novel direction for comprehending the RM sequence by revealing its particular theories in the complex domain, which offer the theoretical bases for constructing RM expansion codes.

### 3.1. Binary RM Codes

For an m×m binary symmetric matrix P∈Sym(m;2) and a binary vector $B\in F_{2}^{m}$, the *n*-th entry of the complexed RM sequence is denoted as

(4) $$C_{P,B}\left(n\right)=\frac{1}{\sqrt{N}}i^{2v_{n-1}^{T}B+v_{n-1}^{T}Pv_{n-1}},\;n=1,\cdots ,N,$$

where *n* is the decimal expression of binary vector $v_{n-1}$, i.e., $\mathrm{decimal}(v_{n-1})=n$. In other words, the RM sequence in (4) is an algebraic equation associated with the time index v, where *n* in $C_{P,B}\left(n\right)$ represents the decimal time index and $v_{n-1}$ is the corresponding binary expression. To facilitate presentation, the index-related RM is illustrated via a simple example and we write out a basis of RM(3,2) as follows [40,41]:

$$\begin{array}{ccccccccc} \left(v_{1},v_{2},v_{3}\right) & \left(1,1,1\right) & \left(1,1,0\right) & \left(1,0,1\right) & \left(1,0,0\right) & \left(0,1,1\right) & \left(0,1,0\right) & \left(0,0,1\right) & \left(0,0,0\right)\\ A=\{2,1\} & 1 & 1 & 0 & 0 & 0 & 0 & 0 & 0\\ A=\{3,1\} & 1 & 0 & 1 & 0 & 0 & 0 & 0 & 0\\ A=\{3,2\} & 1 & 0 & 0 & 0 & 1 & 0 & 0 & 0\\ A=\{1\} & 1 & 1 & 1 & 1 & 0 & 0 & 0 & 0\\ A=\{2\} & 1 & 1 & 0 & 0 & 1 & 1 & 0 & 0\\ A=\{3\} & 1 & 0 & 1 & 0 & 1 & 0 & 1 & 0\\ A=\emptyset & 1 & 1 & 1 & 1 & 1 & 1 & 1 & 1 \end{array}$$

The first row lists all indices $v\in F_{2}^{m}$ for each bit position within a codeword, which can also be denoted as (decimal(1,1,1),decimal(1,1,0),⋯,decimal(0,0,0)) or directly (8,7,⋯,1). Binary Reed–Muller codes consist of the evaluation vectors of multivariate polynomials over the binary field $F_{2}$. The codeword RM(m,r) with parameters *m* and *r* consist of all the evaluation vectors of polynomials with *m* variables and degree no larger than *r*. The second to the last rows are polynomials of degree two, degree one and zero, respectively. It is noteworthy that the first-order part coincides with the first line (bit position indices) and the above basis is the generation matrix for RM(3,2).

### 3.2. Geometry of Complex RM Sequence

Complex RM sequences are made of two parts: a mask sequence [28] $m_{S}={\left[i^{v^{T}Pv}\right]}_{v\in F_{2}^{m}}$ and a Hadamard sequence $h_{B}={\left[i^{2v^{T}B}\right]}_{v\in F_{2}^{m}}$. Overall, the exponents in (4) are evaluations (modulo 4) of degree 2 polynomials in *m* variables and therefore the collection is just an exponentiated second-order Reed–Muller code. However, from a geometric perspective, it is not sufficient to consider only the first- and second-order polynomials. This subsection offers a novel interpretation of RM in the complex domain.

As a matter of fact, for Pauli matrices $E\left(x,y\right)$ with $x,y\in F_{2}^{m}$, the absolute value of $C_{P,B}^{H}E\left(x,y\right)C_{P,B}$ cannot exceed 1 (see more details in [42,43,44]). To proceed, we define a notion associated with shift $e_{r}$ for the Reed–Muller sequence as $C_{P,B}$:

(5) $$F_{A}\left(y\right)=C_{P,B}^{H}E\left(e_{r},y\right)C_{P,B},$$

where 0≤r≤m, $e_{r}$ is the standard basis vector in *m*-dimensional space. To conclude, we summarize the $C_{P,B}$ related geometry theorem in Theorem 1.

**Theorem** **1.** *$C_{P,B}^{H}E\left(e_{r},y\right)C_{P,B}$ is equivalent to implementing a Walsh–Hadamard transform (WHT) on RM sequence $C_{P,B}$.*

**Proof of Theorem 1.**  The formula $C_{P,B}^{H}E\left(e_{r},y\right)C_{P,B}$ can be extended as follows:

(6) $$\begin{array}{c} \begin{array}{cc} F_{A}\left(y\right) & =C_{P,B}^{H}E\left(e_{r},y\right)C_{P,B}\\ & =C_{P,B}^{H}\cdot \left[i^{e_{r}^{T}y}\sum_{a\in F_{2}^{m}}{\left(-1\right)}^{a^{T}y}e_{a+e_{r}}\cdot e_{a}^{T}\right]\cdot C_{P,B}\\ & =\frac{i^{e_{r}^{T}}y}{N}\cdot \sum_{a\in F_{2}^{m}}C_{P,B}^{H}\cdot \left[{\left(-1\right)}^{a^{T}y}\cdot D\left(e_{r},0\right)e_{a}\cdot e_{a}^{T}\right]\cdot C_{P,B}\\ & =\frac{i^{e_{r}^{T}y}}{N}\sum_{a\in F_{2}^{m}}\left[C_{P,B}^{H}\cdot D\left(e_{r},0\right)\right]\cdot \left[D\left(0,y\right)\cdot C_{P,B}\right]\\ & =\frac{i^{e_{r}^{T}y}}{N}\sum_{a\in F_{2}^{m}}\bar{C_{P,B}\left(a+e_{r}\right)}\cdot {\left(-1\right)}^{y^{T}a}\cdot C_{P,B}\left(a\right)\\ & =\frac{i^{e_{r}^{T}y}}{N}\sum_{a\in F_{2}^{m}}{\left(-1\right)}^{{\left(Pe_{r}+y\right)}^{T}a}, \end{array} \end{array}$$

where $N=2^{m}$. The result of (6) completes the proof.    □

In fact, the Hadamard transformations of “shift”, “multiplication” and “shift and multiplication” in Equation (6) show that the process is actually a translation of the “shift-and-multiply” technique into the operation of the Pauli matrices. In essence, this transformation constitutes the difference between the traditional binary RM sequence and the complex one.

The complex RM sequence $C_{P,B}$ still has the following theorem.

**Theorem** **2.** *There is a relationship between between $P_{r}$ and $B_{r}$ as follows:*

(7) $$E\left(e_{r},P_{r}\right)C_{P,B}={\left(-1\right)}^{B_{r}}\cdot C_{P,B},$$

*where $P_{r}=P\cdot e_{r}$ and $B_{r}$ is the r-th element of vector B.*

**Proof of Theorem 2.**  (8) $$\begin{array}{c} \begin{array}{cc} E\left(e_{r},P_{r}\right)C_{P,B} & =\left[i^{e_{r}^{T}P_{r}}\sum_{v\in F_{2}^{m}}{\left(-1\right)}^{P_{r}^{T}v}\cdot e_{v+e_{r}}\cdot e_{v}^{T}\right]\left[\sum_{a\in F_{2}^{m}}i^{a^{T}\mathrm{Pa}+2a^{T}B}\cdot e_{a}\right]\\ & =\frac{i^{P_{r,r}}}{\sqrt{N}}\sum_{v\in F_{2}^{m}}{\left(-1\right)}^{P_{r}^{T}v}\cdot i^{v^{T}Pv+2v^{T}B}\cdot e_{v+e_{r}}\\ \; & =\frac{i^{P_{r,r}}}{\sqrt{N}}\sum_{v\in F_{2}^{m}}{\left(-1\right)}^{{\left(e_{r}+v\right)}^{T}P_{i}}\cdot i^{{\left(e_{r}+v\right)}^{T}P\left(e_{r}+v\right)+2B^{T}\left(e_{r}+v\right)}\cdot e_{v}\\ \; & =\frac{1}{\sqrt{N}}\sum_{v\in F_{2}^{m}}i^{2e_{r}^{T}P_{r}+2P_{r}^{T}v+e_{r}^{T}Pe_{r}+e_{r}^{T}Pv+v^{T}Pe_{r}+v^{T}Pv+2B_{i}+2B^{T}v}\cdot e_{v}\\ \; & =\frac{1}{\sqrt{N}}\sum_{v\in F_{2}^{m}}i^{2B_{r}}i^{3P_{r,r}}i^{4v^{T}Pe_{r}+2B^{T}v+v^{T}Pv}\cdot e_{v}\\ \; & =\frac{{\left(-1\right)}^{B_{r}}}{\sqrt{N}}\sum_{v\in F_{2}^{m}}i^{v^{T}Pv+2B^{T}v}\cdot e_{v}\\ \; & ={\left(-1\right)}^{B_{r}}\cdot C_{P,B} \end{array} \end{array}$$

where $P_{r,r}=e_{r}^{T}P_{r}=e_{r}^{T}Pe_{r}$ and $B_{r}=e_{r}^{T}B$. This completes the proof.    □

Pauli matrices are in one-to-one correspondence to a set of half-space projection operators. Thus, from an estimated $E\left(e_{r},P_{r}\right)$, a projection operator

(9) $$\lambda_{RM,\varpi }^{\left(r\right)}=\frac{\left[I_{N}+\varpi \cdot E\left(e_{r},P_{r}\right)\right]}{2},$$

can be constructed, for which we have

(10) $$\lambda_{RM,\varpi }^{\left(r\right)}C_{P,B}=\left\{\begin{array}{cc} C_{P,B},\; & \;\mathrm{if}\;\varpi ={\left(-1\right)}^{B_{r}},\\ 0, & \;\mathrm{if}\;\varpi \neq {\left(-1\right)}^{B_{r}}. \end{array}\right.$$

## 4. Expansion of Complex Reed–Muller Sequences

In this section, an alternative code of binary subspace chirp (BSSC) in [39] is proposed, which simplifies the number of subspaces in $F_{2}^{m}$, preserves the “patterned” behavior and ultimately exhibits less interference than BSSCs. A geometric feature of the proposed code is also demonstrated, which provides the theoretical basis for the proposed decoding algorithm. We finally propose a projective decoder based on its geometry theorem.

### 4.1. Patterned Reed–Muller Codes

The novel patterned Reed–Muller (PRM) codeword introduces a new parameter Υ that signifies all possible subsets of *m*. Specifically, all potential subsets for *m* of rank Υ∈{1,2,⋯,m} are denoted as χ⊆[m]. Thus, the possible count χ=mΥ varies with variable Υ. The first-order item of the RM generation matrix (mentioned in Section 3.1) is employed here as the position indices $v\in F_{2}^{m}$. In order to retrieve the PRM sequence, it is necessary to manipulate the position indices in a precise manner: first, place “0” bits in all positions but $I_{\chi }v+\tilde{I_{\chi }}{B|}_{\Upsilon +1}^{m}$, where ${B|}_{\Upsilon +1}^{m}$ denotes the sub-sequence of post-(m−Υ) bits of vector B and  $I_{\chi }$ is a matrix of size m×Υ with column vectors $e_{\chi (1)},e_{\chi (2)},\cdots ,e_{\chi (\Upsilon )}$, and $e_{\chi (r)}$ is the unit vector with non-zero at the *r*-th position(1≤r≤Υ). Accordingly, matrix $\tilde{I_{\chi }}$ is of size m×(m−Υ) with column vectors $e_{\tilde{\chi }\left(1\right)},e_{\tilde{\chi }\left(2\right)}\cdots e_{\tilde{\chi }\left(m-\Upsilon \right)}$, where $\tilde{\chi }$ is the complement set of χ in *m*. Second, a sequence RM(Υ,2) is then added to the above non-zero positions and written as follows:

(11) $$C_{\hat{P},B,I_{\chi }}\left(v\right)=\left\{\begin{array}{cc} \frac{1}{\sqrt{2^{\Upsilon }}}i^{2w^{T}{B|}_{1}^{\Upsilon }+w^{T}\hat{P}w},\; & \;\mathrm{if}\;v=I_{\chi }w+\tilde{I_{\chi }}{B|}_{\Upsilon +1}^{m},\\ 0,\; & \;\mathrm{otherwise},\; \end{array}\right.$$

where $w\in F_{2}^{\Upsilon }$ and $\hat{P}$ is the symmetric matrix for RM(Υ,2).

As a matter of fact, the PRM sequence simply reduces the subspace matrix $H_{\tau }$ to $I_{\chi }$; we refer the reader to [39] for more details. Let us further define the matrix $Q=[I_{\chi }\tilde{I_{\chi }}]$ and write (11) in the following format:

(12) $$C_{\hat{P},B,I_{\chi }}\left(v\right)=\frac{{\left(-1\right)}^{\mathrm{wt}\left(B{|}_{\Upsilon +1}^{m}\right)}}{\sqrt{2^{\Upsilon }}}i^{2B^{T}\left(Q^{-1}v\right)+{\left(Q^{-1}v\right)}^{T}P\left(Q^{-1}v\right)}\cdot \left[\phi \left(B,Q^{-1}v,\Upsilon \right)\right],$$

in the case of ${B|}_{\Upsilon +1}^{m}=Q^{-1}{v|}_{\Upsilon +1}^{m}$, $\phi \left(B,Q^{-1}v,\Upsilon \right)$ equal to 1.

**Figure 1:**
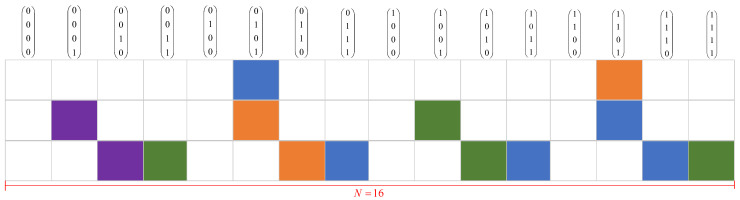
The top line is the indexes of bit position. White = 0, Blue = 1, Purple = −1, Orange= *i*, Green = −i.

We now depict the PRM sequence with a toy example before concluding its general rule. Let m=4, the vector B=[0101] and matrix $S=\left[\begin{array}{cccc} 1 & 1 & 0 & 0\\ 1 & 1 & 1 & 1\\ 0 & 1 & 1 & 0\\ 0 & 1 & 0 & 0 \end{array}\right]$ are fixed; on this basis, three different dimensional spaces of Υ=1,Υ=2 and Υ=3 in $F_{2}^{m}$ with the corresponding subsets χ∈{1}, χ∈{12} and  χ∈{124} are substituted into (11). The resulting sequences are depicted in Figure 1.

We conclude the PRM sequences with the following properties:

- The evidence demonstrates that, given a sequence of length $N=2^{m}$, PRM codes may increase the origin capacity of ${\sqrt{N}}^{\log_{2}N+3}$ while maintaining the code distance and the cardinality of the PRM set Γ is equal to
  (13) |Γ|=∑Υ=1mmΥ·2m·2Υ(Υ+1)2;
- In PRM sequence construction, the RM(Υ,2) is not just inserted into a $2^{m}$-length sequence, but has a sign ${\left(-1\right)}^{\mathrm{wt}\left(B{|}_{\Upsilon +1}^{m}\right)}$ attached (Equation (12) illustrates this). The sign comes from the $i^{2B^{T}\left(Q^{-1}v\right)}$ part, i.e.,  the constraint of $\phi \left(B,Q^{-1}v,\Upsilon \right)$ means the post-(m−Υ) bits should be equal, which leads to the two identical vector multiplication (modulo 4) equal to the weight of ${B|}_{\Upsilon +1}^{m}$ or $\left(Q^{-1}v\right){|}_{\Upsilon +1}^{m}$;
- The vector ${B|}_{1}^{\Upsilon }$ and matrix P serve as the determinants of the non-zero RM(Υ,2) part, where only the upper matrix $P_{\Upsilon }$ is valid. Moreover, the subspace $I_{\chi }$ and ${B|}_{\Upsilon +1}^{m}$ determine the “patterned” form.

### 4.2. Geometry Property of PRM sequence

This section discusses the geometry property of PRM along the lines of Section 3.2 and summarizes the conclusion in Theorem 3.

**Theorem** **3.** *The PRM sequence fulfills the following equation for any $z\in F_{2}^{m-\Upsilon }$:*

(14) $$E\left(I_{\chi }e_{r},\tilde{I_{\chi }}z+I_{\chi }\hat{P}e_{r}\right)\cdot C_{\hat{P},B,I_{\chi }}={\left(-1\right)}^{B^{T}\left(e_{r};0^{m-\Upsilon }\right)+{\left(B{|}_{\Upsilon +1}^{m}\right)}^{T}z}\cdot C_{\hat{P},B,I_{\chi }},$$

*where Υ is dimension of the set χ and $e_{r}$ denotes the unit vector on domain $F_{2}^{\Upsilon }$, 0≤r≤Υ, while $\hat{P}$ is a (Υ×Υ)-element symmetric binary matrix of rank Υ.*

**Proof of Theorem 3.**  The left term of Equation (14) can be expanded as

(15) $$\begin{array}{c} \begin{array}{cc} \; & E\left(I_{\chi }e_{r},{\tilde{I}}_{\chi }z+I_{\chi }\hat{P}e_{r}\right)C_{\hat{P},B,I_{\chi }}\\ \overset{\left(a\right)}{=} & 1/H\cdot i^{{\left(I_{\chi }e_{r}\right)}^{T}\left({\hat{I}}_{\chi }z+I_{\chi }\hat{P}e_{r}\right)}\cdot \sum_{w\in F_{2}^{m}}{\left(-1\right)}^{{\left({\tilde{I}}_{\chi }z+I_{\chi }\hat{P}e_{r}\right)}^{T}w}e_{w+I_{\chi }e_{r}}^{T}e_{w}\\ \; & \cdot \sum_{a\in F_{2}^{m}}i^{{\left(Q^{-1}a\right)}^{T}PQ^{-1}a+2B^{T}Q^{-1}a}\;\phi \left[B,Q^{-1}a,\Upsilon \right]e_{a}\\ = & 1/H\cdot i^{P_{r,r}}\sum_{a\in F_{2}^{m}}{\left(-1\right)}^{{\left({\tilde{I}}_{\chi }z+I_{\chi }\hat{P}e_{r}\right)}^{T}a}\cdot i^{a^{T}Q^{-T}PQ^{-1}a+2B^{T}Q^{-1}a}\cdot \phi \left[B,Q^{-1}a,\Upsilon \right]e_{a+I_{\chi }e_{r}}\\ \overset{\left(b\right)}{=} & 1/H\cdot i^{P_{r,r}}\sum_{a\in F_{2}^{m}}i^{{\underset{︸}{2{\left({\tilde{I}}_{\chi }z+I_{\chi }\hat{P}e_{r}\right)}^{T}\left(a+I_{\chi }e_{r}\right)}}_{A}}\cdot i^{{\underset{︸}{{\left(a+I_{\chi }e_{r}\right)}^{T}Q^{-T}PQ^{-1}\left(a+I_{\chi }e_{r}\right)}}_{B}}\\ & \;\;\;\cdot \underset{C}{\underset{︸}{\phi \left[B,Q^{-1}\left(a+I_{\chi }e_{r}\right),\Upsilon \right]}}\cdot e_{a}, \end{array} \end{array}$$

where $H=2^{\Upsilon }$. The equals sign (a) is based on the formula for Pauli matrices (see Equation (14) in [38] for details) and (b) is the process of replacing all variable a with $\left(a+I_{\chi }e_{r}\right)$. Next, we will develop each item A,B and *C* in the last equation of (15).

Using process ${\left(I_{\chi }\right)}^{T}\cdot I_{\chi }=I_{\Upsilon }$, we expand item *A* into the following equation

(16) $$A=2{\left({\tilde{I}}_{\chi }z+I_{\chi }\hat{P}e_{r}\right)}^{T}a+2P_{r,r},$$

where $P_{r,r}$ is the r-th diagonal element (from top down) of the matrix $\hat{P}$. We further employ the equation $Q^{-1}\cdot I_{\chi }={\left(I_{\chi }\right)}^{T}\cdot I_{\chi }$ and ${\left({\tilde{I}}_{\chi }\right)}^{T}\cdot I_{\chi }=0_{\Upsilon \times \Upsilon }$ and item *B* can be expressed as

(17) $$B={\left(Q^{-1}a\right)}^{T}P\left(Q^{-1}a\right)+2BQ^{-1}a+2{\left({\hat{P}}_{r};0_{m\times r}\right)}^{T}Q^{-1}a+P_{r,r}+2B^{T}\left(e_{r};0^{m-r}\right),$$

where ${\hat{P}}_{r}=\hat{P}e_{r}$. Similarly, we have

(18) $$C=\phi \left[B,Q^{-1}a+\left(e_{r};0^{m-r}\right),\Upsilon \right].$$

The following equation can be obtained by substituting (16)–(18) into (15):

(19) $$\begin{array}{c} \begin{array}{cc} \; & 1/H\cdot i^{4P_{r,r}}{\left(-1\right)}^{B^{T}\left(e_{r};0^{m-r}\right)}\\ & \cdot \sum_{a\in F_{2}^{m}}i^{2{\left({\tilde{I}}_{\chi }z+I_{\chi }\hat{P}e_{r}\right)}^{T}a}\cdot i^{2{\left({\hat{P}}_{r};0_{m\times r}\right)}^{T}Q^{-1}a}\cdot i^{{\left(Q^{-1}a\right)}^{T}P\left(Q^{-1}a\right)+2BQ^{-1}a}\cdot \phi \left[B,Q^{-1}a+\left(e_{r};0^{m-r}\right),\Upsilon \right]\cdot e_{a}\\ \overset{\left(c\right)}{=} & 1/H\cdot {\left(-1\right)}^{B^{T}\left(e_{r};0^{m-r}\right)}\sum_{a\in F_{2}^{m}}i^{2{\left({\tilde{I}}_{\chi }z\right)}^{T}a}\cdot i^{{\left(Q^{-1}a\right)}^{T}P\left(Q^{-1}a\right)+2BQ^{-1}a}\cdot \phi \left[B,Q^{-1}a+\left(e_{r};0^{m-r}\right),\Upsilon \right]\cdot e_{a}\\ \overset{\left(d\right)}{=} & 1/H\cdot {\left(-1\right)}^{B^{T}\left(e_{r};0^{m-r}\right)+{\left(B{|}_{\Upsilon +1}^{m}\right)}^{T}z}\sum_{I_{\chi }w+{\tilde{I}}_{\chi }{B|}_{\Upsilon +1}^{m}\in F_{2}^{m}}i^{{\left(w;B{|}_{\Upsilon +1}^{m}\right)}^{T}\hat{P}\left(w;B{|}_{\Upsilon +1}^{m}\right)+2B^{T}\left(w;B{|}_{\Upsilon +1}^{m}\right)}\cdot e_{I_{\chi }w+{\tilde{I}}_{\chi }{B|}_{\Upsilon +1}^{m}}\\ \overset{\left(e\right)}{=} & {\left(-1\right)}^{B^{T}\left(e_{r};0^{m-r}\right)+{\left(B{|}_{\Upsilon +1}^{m}\right)}^{T}z}\cdot C_{\hat{P},B,I_{\chi }}, \end{array} \end{array}$$

where the equals sign (c) uses the equation $i^{2{\left({\tilde{I}}_{\chi }z+I_{\chi }\hat{P}e_{r}\right)}^{T}a}\cdot i^{2{\left({\hat{P}}_{r};0_{m\times r}\right)}^{T}Q^{-1}a}=i^{2{\left({\tilde{I}}_{\chi }z\right)}^{T}a}$, while (d) replacing a with $I_{\chi }w+{\tilde{I}}_{\chi }{B|}_{\Upsilon +1}^{m}$ as well as using the following formula

(20) $$\begin{array}{c} \begin{array}{cc} \; & \phi \left[B,Q^{-1}a+\left(e_{r};0^{m-r}\right),\Upsilon \right]\\ = & \phi \left[B,Q^{-1}\left(I_{\chi }w+{\tilde{I}}_{\chi }B{|}_{\Upsilon +1}^{m}\right)+\left(e_{r};0^{m-r}\right),\Upsilon \right]\\ = & \phi \left[B,\left(w;B_{1}^{\Upsilon }\right)+\left(e_{r};0^{m-r}\right),\Upsilon \right]\\ = & \phi \left[B,(w+e_{r};B_{1}^{\Upsilon }),\Upsilon \right]=1 \end{array} \end{array}$$

to eliminate ϕ(·) as 1. We use the equation

(21) $$\begin{array}{c} \begin{array}{cc} C_{\hat{P},B,I_{\chi }}=\; & \sum_{I_{\chi }w+{\tilde{I}}_{\chi }{B|}_{\Upsilon +1}^{m}\in F_{2}^{m}}i^{{\left(w;B{|}_{\Upsilon +1}^{m}\right)}^{T}\hat{P}\left(w;B{|}_{\Upsilon +1}^{m}\right)+2B^{T}\left(w;B{|}_{\Upsilon +1}^{m}\right)}\cdot e_{I_{\chi }w+{\tilde{I}}_{\chi }{B|}_{\Upsilon +1}^{m}}\\ \overset{\left(f\right)}{=} & \sum_{w\in F_{2}^{\Upsilon }}i^{w^{T}\hat{P}w+2B_{1}^{\Upsilon }+\mathrm{wt}\left(B_{1}^{\Upsilon }\right)}\cdot e_{I_{\chi }w+{\tilde{I}}_{\chi }{B|}_{\Upsilon +1}^{m}} \end{array} \end{array}$$

in (e). Thus, (19) agrees with the conclusion in Theorem 3.    □

**Lemma** **1.** *For any $z\in F_{2}^{m-\Upsilon }$, the following projection operator exists*

(22) $$\lambda_{PRM,\varpi }^{\left(r\right)}\left(z{|}_{z\in F_{2}^{m-\Upsilon }}\right)=\frac{\left[I_{N}+\varpi \cdot E\left(I_{\chi }e_{r},{\left[\tilde{I_{\chi }}z+I_{\chi }\hat{P}e_{r}\right]}_{z\in F_{2}^{m-\Upsilon }}\right)\right]}{2},$$

*where $\varpi ={\left(-1\right)}^{B_{r}+{\left(B{|}_{\Upsilon +1}^{m}\right)}^{T}{z|}_{z\in F_{2}^{m-\Upsilon }}}$. For any vector $\tilde{z}\in F_{2}^{m-\Upsilon }$ we get*

(23) $$\lambda_{PRM,\varpi }^{\left(r\right)}\left(\tilde{z}\right)\cdot C_{\hat{P},B,I_{\chi }}=\left\{\begin{array}{cc} C_{\hat{P},B,I_{\chi }},\; & \;\mathrm{if}\;\varpi ={\left(-1\right)}^{B_{r}+{\left(B{|}_{\Upsilon +1}^{m}\right)}^{T}\tilde{z};},\\ 0,\; & \;\mathrm{if}\;\varpi \neq {\left(-1\right)}^{B_{r}+{\left(B{|}_{\Upsilon +1}^{m}\right)}^{T}\tilde{z}.} \end{array}\right.$$

*If $\tilde{z}=0^{m-\Upsilon }$ is fixed, we have*

(24) $$\lambda_{PRM,\varpi }^{\left(r\right)}\left(0^{m-\Upsilon }\right)=\frac{I_{N}+\varpi \cdot E\left(I_{\chi }e_{r},I_{\chi }\hat{P}e_{r}\right)}{2},$$

*and we get*

(25) $$\lambda_{PRM,\varpi }^{\left(r\right)}\left(0^{m-\Upsilon }\right)\cdot C_{\hat{P},B,I_{\chi }}=\left\{\begin{array}{cc} C_{\hat{P},B,I_{\chi }},\; & \;\mathrm{if}\;\varpi ={\left(-1\right)}^{B_{r};},\\ 0,\; & \;\mathrm{if}\;\varpi \neq {\left(-1\right)}^{B_{r}.} \end{array}\right.$$

Lemma 1 is the core idea of the detection algorithm.

### 4.3. The Proposed Geometry-Based PRM Detection

We model a single-user scenario in which the receiver observes

(26) $$\begin{array}{c} \begin{array}{c} y=C_{\hat{P},B,I_{\chi }}+n, \end{array} \end{array}$$

where $C_{\hat{P},B,I_{\chi }}$ is a PRM sequence while n represents an additive noise. Unlike the RM sequence decoding algorithm, PRM code needs to decode three items, i.e., $\left\{I_{\chi },\hat{P},B\right\}$. In view of the fact that decoding the second-order RM(Υ,2) at non-zero positions can be accomplished only if the subspace $I_{\chi }$ is known, the knowledge of subspace matrix $I_{\chi }$ should be recovered in priority. Algorithm 1 summarizes the detail of the proposed decoding algorithm.
**Algorithm 1:** Estimation of single PRM sequence
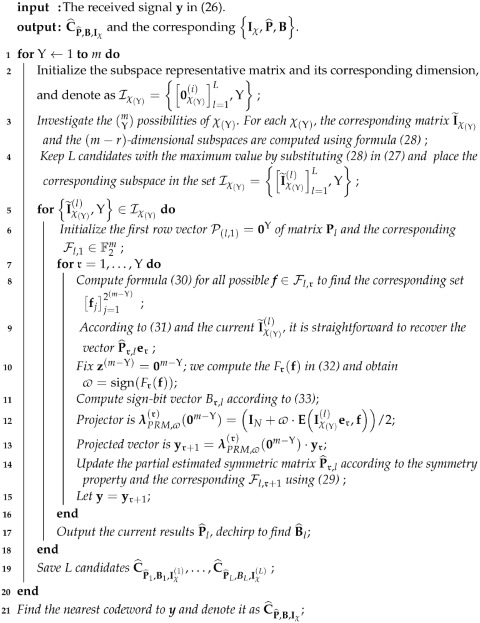


    Due to the indeterminate rank of the received PRM sequence, we traverse all possible ranks 1≤Υ≤m and reserve *L* candidate subspaces beneath each rank (line 1). Lines 2–5 describe the process for collecting candidates: for each vector $f_{\chi_{\left(\Upsilon \right)}}\in F_{2}^{m}$, we compute the related $y^{H}E\left(0,f_{\chi_{\left(\Upsilon \right)}}\right)y$ and its summation form

(27) $$\begin{array}{c} \begin{array}{c} F_{\mathrm{sum}}\left(f_{\chi_{\left(\Upsilon \right)}}\right)=\sum_{j=1}^{2^{\left(m-\Upsilon \right)}}\left|y^{H}E\left(0,f_{\chi_{\left(\Upsilon \right)}}^{\left(j\right)}\right)y\right|. \end{array} \end{array}$$

By searching the largest $2^{\left(m-\Upsilon \right)}$ estimatios of $F_{\mathrm{sum}}$, the corresponding set ${\left[f_{\chi_{\left(\Upsilon \right)}}^{\left(j\right)}\right]}_{j=1}^{2^{\left(m-\Upsilon \right)}}$ and the subspace matrix ${\tilde{I}}_{\chi_{\left(\Upsilon \right)}}$ are identified and placed as a candidate, in which the relation between ${\left[f_{\chi_{\left(\Upsilon \right)}}^{\left(j\right)}\right]}_{j=1}^{2^{\left(m-\Upsilon \right)}}$ and ${\tilde{I}}_{\chi_{\left(\Upsilon \right)}}$ is

(28) $${\left[f_{\chi_{\left(\Upsilon \right)}}^{\left(j\right)}\right]}_{j=1}^{2^{\left(m-\Upsilon \right)}}=\mathrm{span}\left({\tilde{I}}_{\chi_{\left(\Upsilon \right)}}\right).$$

We repeat the above steps and keep *L* candidates in set $I_{\chi_{\left(\Upsilon \right)}}=\left\{{\left[{\tilde{I}}_{\chi_{\left(\Upsilon \right)}}^{\left(l\right)}\right]}_{l=1}^{L},\Upsilon \right\}$.

The subsequent RM(Υ,2) recovery is based on the subspaces in candidates $I_{\chi_{\left(\Upsilon \right)}}$. As shown in lines 9–18, the symmetric matrix ${\hat{P}}_{l}$ and the vector $B_{l}$ for decoding RM(Υ,2) are recovered layer by layer within the *l*-th interaction, where the subscript *l* denotes the current *l*-th candidate in $I_{\chi_{\left(\Upsilon \right)}}$. Since the matrix is of Kerdock set with symmetric properties, we denote the set of indexes for the rows determined by decoded layers as R (R=1,⋯,r−1). Utilizing the estimated row vectors to complete the symmetric matrix, the current matrix for the r-th layer decoding can be represented as ${\underline{\hat{P}}}_{R}$. Hence, the search space under the *l*-th submatrix is limited to

(29) $$F_{l,r}=\left\{f\in F_{2}^{m}|f_{i}={\underline{\hat{P}}}_{R}\left(i,r\right)\;\mathrm{for}\;\mathrm{all}\;i\in R\right\},$$

where the value of ${\underline{\hat{P}}}_{R}\left(i,r\right)$ refers to the *i*-th row and r-th column of matrix ${\underline{\hat{P}}}_{R}$. On this basis, for all vectors $f\in F_{l,r}$, an equation written as

(30) $$\begin{array}{c} \begin{array}{c} F_{\mathrm{sum}}\left(f\right)=\sum_{j=1}^{2^{\left(m-\Upsilon \right)}}\left|y^{H}E\left(I_{\chi_{\left(\Upsilon \right)}}^{\left(l\right)}\cdot e_{r},f\right)y\right| \end{array} \end{array}$$

is calculated to search the largest $2^{\left(m-\Upsilon \right)}$ values of $F_{\mathrm{sum}}\left(f\right)$ and herein find the corresponding set ${\left[f_{j}\right]}_{j=1}^{2^{\left(m-\Upsilon \right)}}$ based on

(31) $$\begin{array}{c} \begin{array}{c} {\left[f_{j}\right]}_{j=1}^{2^{\left(m-\Upsilon \right)}}=\mathrm{span}\left({\tilde{I}}_{\chi_{\left(\Upsilon \right)}}^{\left(l\right)}\right)+I_{\chi_{\left(\Upsilon \right)}}^{\left(l\right)}{\hat{P}}_{r,l}e_{r}. \end{array} \end{array}$$

We finally get ${\hat{P}}_{r,l}e_{r}$, where ${\hat{P}}_{r,l}$ is the r-th row/column vector for ${\hat{P}}_{l}$.

In the case of $B_{l}$ decoding, as can be seen from Lemma 1, we first compute $F_{r}\left(f\right)$ by substituting (31) into (30) and letting $z^{\left(m-\Upsilon \right)}=0^{m-\Upsilon }$:

(32) $$\begin{array}{c} \begin{array}{c} F_{r}\left(f\right)=y^{H}E\left(I_{\chi_{\left(\Upsilon \right)}}^{\left(l\right)}\cdot e_{r},I_{\chi_{\left(\Upsilon \right)}}^{\left(l\right)}{\hat{P}}_{r,l}e_{r}\right)y, \end{array} \end{array}$$

we then obtain $\mathrm{sign}\left(F_{r}\left(f\right)\right)={\left(-1\right)}^{B_{r,l}}$. Therefore, $B_{r}$ can be obtained immediately as

(33) $$B_{r,l}=\left\{\begin{array}{cc} 1,\; & \;\mathrm{if}\;\mathrm{sign}\left(F_{r}\left(f\right)\right)=-1;\\ 0,\; & \;\mathrm{if}\;\mathrm{sign}\left(F_{r}\left(f\right)\right)=1. \end{array}\right.$$

(33) can be summarized as the relationship of

(34) $$B_{r,l}=\frac{\left(1-\varpi \right)}{2},$$

where $\varpi =\mathrm{sign}\left(F_{r}\left(f\right)\right)$.

The power of y (subscripts for r=1 is omitted) projected with this operator is

(35) $$\begin{array}{c} \begin{array}{cc} \; & {∥\lambda_{PRM,\varpi }\left(0^{m-\Upsilon }\right)\cdot y∥}^{2}\\ = & y^{H}\cdot \lambda_{PRM,\varpi }\left(0^{m-\Upsilon }\right)\cdot y\\ = & \frac{1}{2}[C_{\hat{P},B,I_{\chi }}^{H}C_{\hat{P},B,I_{\chi }}+\varpi C_{\hat{P},B,I_{\chi }}^{H}E\left(I_{\chi_{\left(\Upsilon \right)}}^{\left(l\right)}e_{r},f\right)C_{\hat{P},B,I_{\chi }}+\varpi C_{\hat{P},B,I_{\chi }}^{H}E\left(I_{\chi_{\left(\Upsilon \right)}}^{\left(l\right)}e_{r},f\right)n+C_{\hat{P},B,I_{\chi }}^{H}n\\ & +\varpi n^{H}E\left(I_{\chi_{\left(\Upsilon \right)}}^{\left(l\right)}e_{r},f\right)C_{\hat{P},B,I_{\chi }}+\varpi n^{H}E\left(I_{\chi_{\left(\Upsilon \right)}}^{\left(l\right)}e_{r},f\right)n+C_{\hat{P},B,I_{\chi }}^{H}n+n^{H}n]\\ \geq & \frac{1}{2}\left[C_{\hat{P},B,I_{\chi }}^{H}C_{\hat{P},B,I_{\chi }}+\varpi \cdot F_{r}\left(f\right)\right], \end{array} \end{array}$$

where $f=I_{\chi_{\left(\Upsilon \right)}}^{\left(l\right)}{\hat{P}}_{r,l}e_{r}$. We see that the halfspace to which the projection of y is largest.

We perform a projection operation on the r-th layer signal y before performing the next layer decoding by employing the projection operator $\lambda_{DRM,\varpi }^{\left(r\right)}\left(0^{m-\Upsilon }\right)$ (see line 14 in Algorithm 1). While 2≤r≤Υ, the input signal becomes

(36) $$\begin{array}{c} \begin{array}{cc} \; & y_{r}=\lambda_{PRM,\varpi }^{\left(r\right)}\cdot y_{r-1}\\ = & C_{\hat{P},B,I_{\chi }}+\prod_{i=1}^{r-1}\lambda_{PRM,\varpi }^{\left(i\right)}\cdot n\\ = & C_{\hat{P},B,I_{\chi }}+\frac{1}{2^{r-1}}\prod_{i=1}^{r-1}\left(I_{N}+\varpi_{i}\cdot E\left[I_{\chi }e_{i},I_{\chi }\hat{P}e_{i}\right]\right)\cdot n, \end{array} \end{array}$$

which indicates that the power of noise is halved within each iteration.

Line 21 evaluates the *L* results under each rank and retains the optimal solution. Finally, line 23 selects the optimal solution for the results of all ranks.

## 5. Unsourced Random Access Scheme Using Patterned Reed–Muller Sequence

The *k*-th active user preferentially converts its message sequence $U^{\left(k\right)}$ into the corresponding *M*-length *J*-ary Reed–Solomon code, which completes the outer A-channel coding. Before conducting the inner encoding, a random prefix message is repeatedly added into *M* sub-blocks. The combination of the prefix and *J*-ary bits can produce a *G*-ary message in each slot. By using this prefix, an slot occupation criterion is selected from a common pool Ω. The binary sequence transformed by a *G*-ary bit is subsequently mapped to an element in a common codebook Γ and the resulting codeword is then transmitted to the AP side. In the receiver, the AP detects the users’ messages using the proposed projection method. In the sequel, we will describe every module of the proposed URA system in detail.

### 5.1. Transmitter

**Figure 2:**
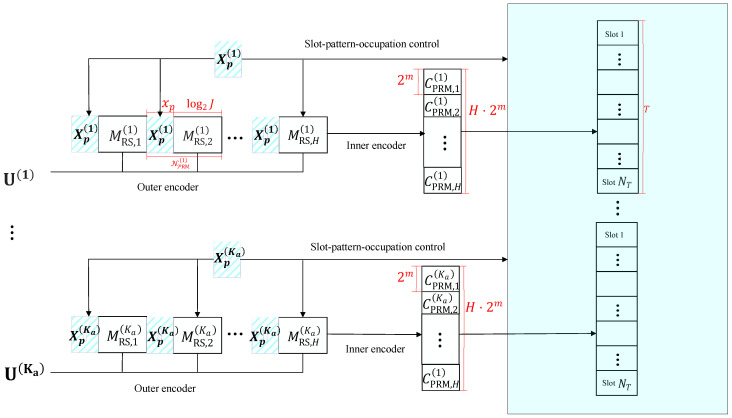
The diagram of the transmitter technique in our proposed URA system.

The specific structure of the transmitter is shown in Figure 2. This subsection provides a detailed description of how the transmission sequence is generated in the transmitter, followed by the construction of the slot-pattern-control pool.

#### 5.1.1. Transmitter Design

The A-channel code used for the outer encoding is derived from paper [22]: the *k*th user’s message $U^{\left(k\right)}$ of length *B* is mapped to a *J*-ary Reed–Solomon code of length *H*, designated ${\left[{\underline{M}}_{\mathrm{RS},h}^{\left(k\right)}\right]}_{h=1}^{H}=\left(M_{\mathrm{RS},1}^{\left(k\right)},M_{\mathrm{RS},2}^{\left(k\right)},\cdots ,M_{\mathrm{RS},H}^{\left(k\right)}\right)\in {\left[J\right]}^{H}$, 0≤h≤H, where $M_{\mathrm{RS},h}^{\left(k\right)}\in \left[J\right]$ is the *h*-th output bit of outer coding. To satisfy the coding rate requirement, we repeatedly append the binary prefix sequence $X_{p}^{\left(k\right)}$ to each *J*-ary bit $M_{\mathrm{RS},h}^{\left(k\right)}$ to create the *G*-ary sequence. Thus, the length of the binary sequence $X_{p}^{\left(k\right)}$ is set to $\log_{2}\left(G/J\right)$ and we denote $x_{p}=\log_{2}\left(G/J\right)$. For further interpretation, a *G*-ary bit is divided into $2^{x_{p}}$ cosets, each containing $2^{J}$ components, and this process can be summarized as follows:

(37) $${\overset{˘}{m}}_{\mathrm{PRM},h}^{\left(k\right)}=ϝ\left(\left[X_{p}^{\left(k\right)};M_{\mathrm{RS},h}^{\left(k\right)}\right]\right),$$

where 𝘍(·) is the bijective mapping: $𝘍\left(\cdot \right):\left[2^{x_{p}}\right]\times \left[J\right]$ and ${\overset{˘}{m}}_{\mathrm{PRM},h}^{\left(k\right)}\in G$. By repeating (37) *H* times, an *H*-length sequence ${\left[{\underline{m}}_{\mathrm{PRM},h}^{\left(k\right)}\right]}_{h=1}^{H}=\left({\overset{˘}{m}}_{\mathrm{PRM},1}^{\left(k\right)},{\overset{˘}{m}}_{\mathrm{PRM},2}^{\left(k\right)},\cdots ,{\overset{˘}{m}}_{\mathrm{PRM},H}^{\left(k\right)}\right)$ is obtained, which serves as the input sequence for the inner encoder. We require a one-to-one matching between ${\overset{˘}{m}}_{\mathrm{PRM}}^{\left(k\right)}$ and codeword $C_{\mathrm{PRM}}^{\left(k\right)}$ from the common codebook (we omit the subscript *m* here for simplicity), and, herein, ${\overset{˘}{m}}_{\mathrm{PRM}}^{\left(k\right)}$ is the input signal of the inner encoder while the output codeword is $C_{\mathrm{PRM}}^{\left(k\right)}$.

Let the message ${\overset{˘}{m}}_{\mathrm{PRM}}^{\left(k\right)}$ have a length of $N_{\mathrm{PRM},h}^{\left(k\right)}$; it is evident that $N_{\mathrm{PRM}}^{\left(k\right)}=\log_{2}G$. Furthermore, according to the codebook capacity |Γ| of Equation (13), the size of information bits that a PRM sequence can carry in each slot for user *k* is $N_{\mathrm{PRM}}^{\left(k\right)}=\left\lfloor \log_{2}\left(|\Gamma |\right)\right\rfloor$. Finally, the output of inner coding over *H* slots is recorded as ${\left[{\underline{C}}_{\mathrm{PRM},h}^{\left(k\right)}\right]}_{h=1}^{H}=\left(C_{\mathrm{PRM},1}^{\left(k\right)},C_{\mathrm{PRM},2}^{\left(k\right)},\cdots ,C_{\mathrm{PRM},H}^{\left(k\right)}\right)$. This process of the transmitter is depicted in Figure 2.

#### 5.1.2. The Construction of Slot-Pattern-Control Pool

In the practical encoding procedure, each user inserts its *H*-length RS code into $N_{T}$ slots by looking up a criterion from the common dictionary Ω. Specifically, we construct a collection pool Ω for holding the common slot-pattern-control criteria and the *k*-th user retrieves the matching SPC from the pool based on the specific $X_{p}^{\left(k\right)}$.

The slot-pattern-control scheme is designed using a manner that refers to the construction of PRM code as a heuristic algorithm: the initial step is to unify all users’ slot indices, which can be managed by utilizing the binary notation $t\in F_{2}^{\ddot{m}}$ to identify each slot. It is evident that, for a time slot of length $N_{T}$, the equation $N_{T}=2^{\ddot{m}}$ is necessary. Next, we extend the subspace generation rule in $F_{2}^{m}$ for the PRM construction to classify $N_{T}$ time slots into several sub-slots; in detail, let $N_{\mathrm{group}}$ denote the possible number of *r* dimensional spaces in $F_{2}^{m}$ with Ngroup=m¨r and the corresponding subset is represented as ξ. Obviously, the length of ξ is *r* and $\xi \in F_{2}^{\ddot{m}}$. On the condition that *r* is constant, the slots used to place the $2^{r}$-length ($H=2^{r}$) outer codeword are labeled as

(38) $${\left[t_{\xi }^{\left(h\right)}\right]}_{h=1}^{H}=I_{\xi }x^{r}+\tilde{I_{\xi }}{b|}_{r+1}^{\ddot{m}}\;x^{r}\in F_{2}^{r},$$

where the set $\left\{I_{\xi }x^{r}\right\}$ (span all $x^{r}\in F_{2}^{r}$) indicates a subspace and the vector $\tilde{I_{\xi }}{b|}_{r+1}^{\ddot{m}}$ controls the cosets of the subspace. Since vector ${b|}_{r+1}^{\ddot{m}}$ has $2^{\left(\ddot{m}-r\right)}$ possibilities, $n_{\mathrm{subgroup}}=2^{\left(\ddot{m}-r\right)}$ cosets exist for each subspace. On this basis, the pool Ω generates a total of Ω=m¨r·2(m¨−r) candidates and the length of the information $X_{p}^{\left(k\right)}$ is fixed as $x_{p}=\left\lfloor \log_{2}\left|\Omega \right|\right\rfloor$. In this way, once the user has acquired the knowledge of $X_{p}^{\left(k\right)}$ (there exists a one-to-one correspondence between $\left\{{b|}_{r}^{\ddot{m}},\xi \right\}$-pair and sequence $X_{p}^{\left(k\right)}$), an *H* length codeword is successively inserted into the slots in accordance with (38).

In general, given a SPC ${\left[t_{\xi }^{\left(h\right)}\right]}_{h=1}^{H}$ and a random slot index $t_{h}\in F_{2}^{m}$, the signals to be sent in slot $t_{h}$ are formulated as

(39) $$x_{k,n}=\left\{\begin{array}{cc} C_{\mathrm{PRM},h}^{\left(k\right)},\; & \;\mathrm{if}\;t_{h}\in {\left[t_{\xi }^{\left(h\right)}\right]}_{h=1}^{H};\\ 0^{N},\; & \;\mathrm{if}\;t_{h}\notin {\left[t_{\xi }^{\left(h\right)}\right]}_{h=1}^{H}. \end{array}\right.$$

We let the vector $t_{h}\left[\xi \right]\in F_{2}^{r}$ retain the certain bits from vector $t_{\xi }^{\left(h\right)}$ at locations of ξ and regard $h=\mathrm{decimal}\left(t_{h}\left[\xi \right]\right)+1$ as the decimal index of non-zero position in all slots. Moreover, $n=\mathrm{decimal}\left(t_{h}\right)+1$, $1\leq n\leq N_{T}$.

### 5.2. Slot-Based PRM Detection and Reed–Solomon List Recovery Decoding

The complete signal sent by the active user *k* is expressed as

(40) $$x_{k}=\left[x_{k,1};\cdots ;x_{k,N_{T}}\right],$$

after receiving the signals, the AP prioritizes performing decoding operations in each slot. To be more specific, the received signal in time slot *n*$\left(1\leq n\leq N_{T}\right)$ can be expressed as

(41) $$y_{n}=\sum_{k=1}^{K_{a}}h_{k,n}\cdot x_{k,n}+n_{n},\;$$

where $h_{k,n}∼CN\left(0,1\right)$ denotes the channel coefficient between the active user *k* and AP in slot *n*. The coefficients $h_{k,n}$ are estimated in the process of identifying the most probable transmitted signals, while $n_{n}∼CN\left(0,I_{N}\right)$ is the complex additive white Gaussian noise (AWGN).

In Algorithm 2, we summarize the process of PRM reconstruction in a multi-user scenario within a single slot. We consider the input signal $Y=(y_{1},\cdots ,y_{N_{T}})$ as a linear superposition of $K_{a}$ users and require the AP to output all the message set $\left\{W_{1},W_{2},\cdots ,W_{K_{a}}\right\}$. Lines 1–7 of Algorithm 2 summarize the OMP-based inner-code detection algorithm in the multi-user scenario. Since OMP is an iterative algorithm, the number of output codewords is equivalent to the count of decoding iterations $K_{0}$. For the outer-decode process, we denote $Y=(Y_{1},\cdots ,Y_{H})$, $\left(Y_{h}\subseteq \left[H\right]\right)$ as the input signal of the outer-decoder, which results from removing the coset sequence from the messages received from inner decoding and connecting the repeated $N_{T}$ results. This process is described in lines 8–10. Lines 12 to 15 perform the outer Reed–Solomon decoding process and the detailed procedure is suggested in the literature [22]. All possibilities of decoding results for each user are saved in the list $V_{L}$.
**Algorithm 2:** PRM reconstruction in a multi-user scenario
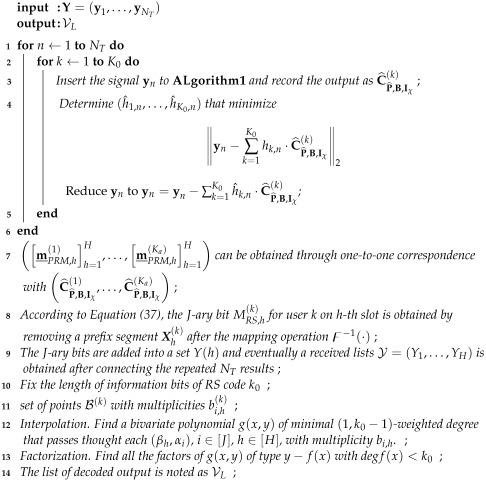


## 6. Performance Analysis

Our proposed URA system depends on the reliability of PRM detection in each chunk and the efficiency of the outer decoder in coupling the information between slots. The purpose of this section is to discuss how the PRM sequence is distributed in each sub-block, which is one of the most crucial factors influencing slot-based inner-code detection performance.

### The PRM Distribution for a Single Slot

The number of sequences for a received signal involved in linear superposition is the key factor governing the distribution of codewords within a particular slot.

Next, we define a random variable T to further explore the above event $\tilde{t_{s}}\in {\left[t_{\xi }^{\left(i\right)}\right]}_{i=1}^{\frac{2^{\ddot{m}}}{n_{\mathrm{subgroup}}}}$ and we use T=1 to denote the event that “a set corresponding to the selected rule contains a specific slot index”.

To examine the probability that the time slot $\tilde{t_{s}}$ will be chosen, evaluate the likelihood that the event “a slot $\tilde{t_{s}}$ is contained in the chosen set ${\left[t_{\xi }^{\left(i\right)}\right]}_{i=1}^{\frac{2^{\ddot{m}}}{n_{\mathrm{subgroup}}}}$” takes place is sufficient, which can be described as $\tilde{t_{s}}\in {\left[t_{\xi }^{\left(i\right)}\right]}_{i=1}^{\frac{2^{\ddot{m}}}{n_{\mathrm{subgroup}}}}$ and the probability equals

(42) $$\mathrm{Pr}\left[\tilde{t_{s}}\in {\left[t_{\xi }^{\left(i\right)}\right]}_{i=1}^{\frac{2^{\ddot{m}}}{n_{\mathrm{subgroup}}}}\right]=\frac{N_{\mathrm{group}}}{2^{\ddot{m}-r}\cdot N_{\mathrm{group}}}=2^{r-\ddot{m}}=n_{\mathrm{subgroup}}^{-1},$$

where Ngroup=m¨r and $n_{\mathrm{subgroup}}=2^{\left(\ddot{m}-r\right)}$. This result is numerically equivalent to the probability that a coset will be selected when a subspace $I_{\xi }$ is provided. As a matter of fact, a specific slot will appear only once in each manner of partitioning the space $F_{2}^{m}$. Thus, with a total of m¨r partition manners, a specific bit will appear m¨r times in all conceivable subsets 2(m¨−r)·m¨r. Furthermore, the number of subgroups is $n_{\mathrm{subgroup}}$ and the exact location is repeated $N_{\mathrm{group}}$ times for a given subspace $I_{\xi }$; herein, users who choose the same subspace but different subgroups will not collide; in other words, for two random selections within a given subspace, the probability that no conflicts will arise equals the chance of selecting two different subsets.

Next, we use T=1 to denote the event “a set corresponding to the selected rule contains a specific slot index”. According to (42), the variable T obeys the Bernoulli distribution; we then derive the expectation $E\left[T\right]=n_{\mathrm{subgroup}}^{-1}$. As the value of |Ω| is $2^{x_{m}}$, the number of probable appearances for slot $\tilde{t_{s}}$ is formulated as

(43) $$N_{t}=2^{x_{p}}\cdot n_{\mathrm{subgroup}}^{-1},$$

which holds for arbitrary locations.

Let the random variable K denote the total number of simultaneous appearances for $K_{a}$ users in slot $\tilde{t_{s}}$. Consider the case in which users select separate elements from pool Ω, which means that all users are not putting elements back while selecting rules. Thus, the probability mass function (PMF) of K is calculated as follows

(44) $${\mathrm{Pr}}_{\mathrm{unrepeatable}}\left[K=\kappa \right]=\frac{\left(\begin{array}{c} N_{t}\\ \kappa \end{array}\right)\left(\begin{array}{c} N_{\mathrm{group}}\cdot n_{\mathrm{subgroup}}-N_{t}\\ K_{a}-\kappa \end{array}\right)}{\left(\begin{array}{c} N_{\mathrm{group}}\cdot n_{\mathrm{subgroup}}\\ K_{a} \end{array}\right)},$$

where $\kappa =1,\cdots ,N_{t}$, $K_{a}=0,\cdots ,N_{\mathrm{group}}\cdot n_{\mathrm{subgroup}}$. Accordingly, the variable K is expected to be

(45) $$E_{\mathrm{unrepeatable}}\left[K\right]=\frac{K_{a}\cdot N_{t}}{N_{\mathrm{group}}\cdot n_{\mathrm{subgroup}}},$$

and variance have the form of

(46) $$D_{\mathrm{unrepeatable}}\left[K\right]=\frac{K_{a}\cdot N_{t}}{N_{\mathrm{group}}\cdot n_{\mathrm{subgroup}}}\frac{N_{\mathrm{group}}\cdot n_{\mathrm{subgroup}}-K_{a}}{N_{\mathrm{group}}\cdot n_{\mathrm{subgroup}}-1}\left(1-\frac{N_{t}}{N_{\mathrm{group}}\cdot n_{\mathrm{subgroup}}}\right).$$

It is assumed that users are not prohibited from selecting the same rules more than once (SPC collisions) and K obeys the PMF being

(47) $${\mathrm{Pr}}_{\mathrm{repeatable}}\left[K=\kappa \right]=\left(\begin{array}{c} K_{a}\\ \kappa \end{array}\right){\left(\frac{N_{t}}{N_{\mathrm{group}}\cdot n_{\mathrm{subgroup}}}\right)}^{\kappa }{\left(1-\frac{N_{t}}{N_{\mathrm{group}}\cdot n_{\mathrm{subgroup}}}\right)}^{K_{a}-\kappa }.$$

K obey the binomial distribution with its expectation and variance expressed as

(48) $$E_{\mathrm{repeatable}}\left[K\right]=\frac{K_{a}\cdot N_{t}}{N_{\mathrm{group}}\cdot n_{\mathrm{subgroup}}},$$

and

(49) $$D_{\mathrm{repeatable}}\left[K\right]=\frac{K_{a}\cdot N_{t}}{N_{\mathrm{group}}\cdot n_{\mathrm{subgroup}}}\left(1-\frac{N_{t}}{N_{\mathrm{group}}\cdot n_{\mathrm{subgroup}}}\right).$$

We find that $E_{\mathrm{unrepeatable}}\left[K\right]=E_{\mathrm{repeatable}}\left[K\right]$, while $D_{\mathrm{unrepeatable}}\left[K\right]=D_{\mathrm{repeatable}}\left[K\right]$. Different strategies for picking rules from pool Ω do not affect the average number of codewords in one slot. Furthermore, when the active users choose from the separate SPCs, the inner codes are distributed more uniformly throughout the slots. In the presence of selecting the same rules more than once, the probability that many PRM sequences could collide in one slot increases, thus resulting in a higher multi-user interference (MUI).

## 7. Simulation Results

### 7.1. The Distribution of Slot-Based PRM Sequences

**Figure 3:**
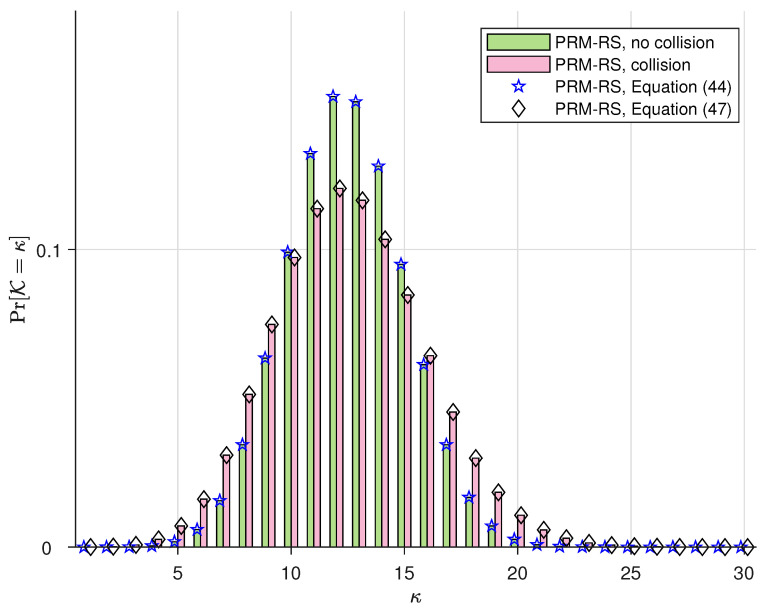
The distribution of PRM sequences across slots for $\ddot{m}=8$, r=5 and m=8 for the benchmark scheme, both under $K_{a}=100$.

**Figure 4:**
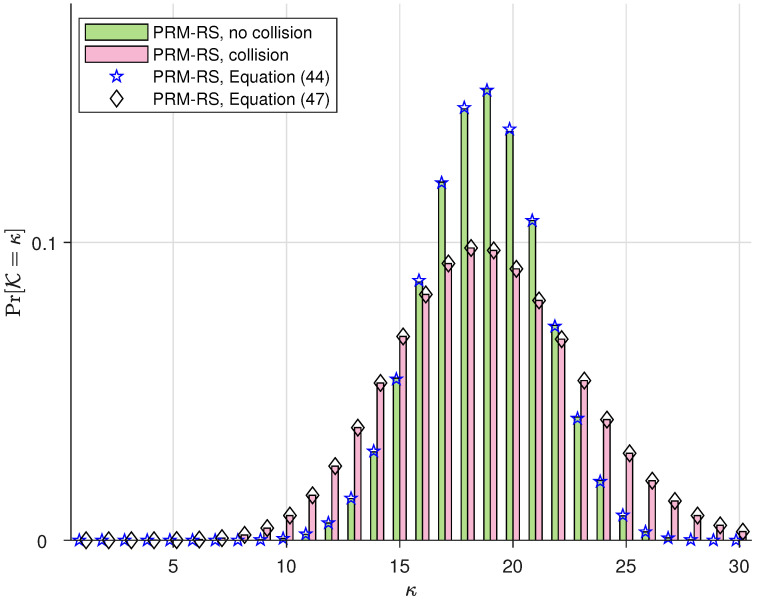
The distribution of PRM sequences across slots for $\ddot{m}=8$, r=5 and m=8 for the benchmark scheme, both under $K_{a}=150$.

**Figure 5:**
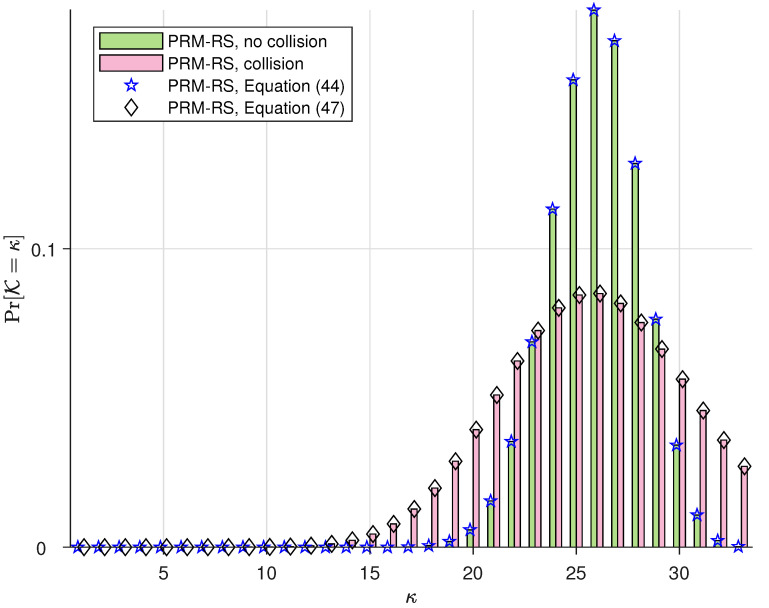
The distribution of PRM sequences across slots for $\ddot{m}=8$, r=5 and m=8 for the benchmark scheme, both under $K_{a}=200$.

**Figure 6:**
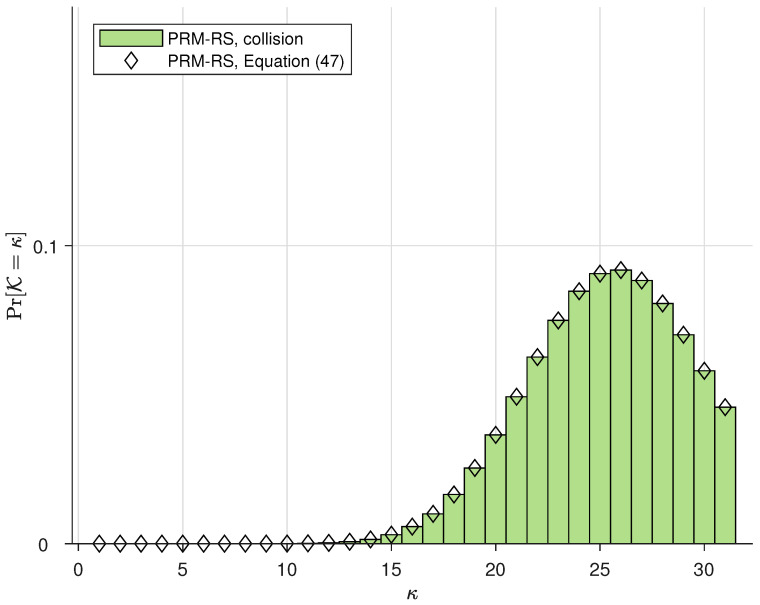
The distribution of PRM sequences across slots for $\ddot{m}=8$, r=6 and $K_{a}=100$.

The simulations in this subsection illustrate the probability distribution of the number of PRM codes that can be accommodated simultaneously in one slot, i.e., Pr(K). We denote “PRM-RS, no collision” and “PRM-RS, collision”, respectively, as the practical simulation results with and without SPC collision. Moreover, we denote “PRM-RS, Equation (44)” and “PRM-RS, Equation (47)”, respectively, as the theoretical results calculated from (44) and (47). The parameters for our PRM-RS scheme are $\ddot{m}=8$ and r=5, and the sequence distributions for $K_{a}=100,150$ and 200 users are, respectively, depicted in Figure 3, Figure 4 and Figure 5. Moreover, Figure 6 illustrates the results with parameters $\ddot{m}=8$ and r=6 under $K_{a}=100$. Observations can be drawn from this as follows:

1. PRM sequences are distributed more evenly when active users employ different SPCs. If collisions occur, more codewords will overlap at one slot, resulting in a rise in multi-user interference (MUI) and an increased probability of failure detection.
2. According to Figure 3, Figure 4 and Figure 5, the distributions of “PRM-RS” and “RM-Shift” almost overlap until $k_{a}=200$. Compared to the benchmark, the overall simulation of the simultaneously accommodated codeword count for “PRM-RS” becomes larger when the number of users exceeds 200.
3. Figure 6 illustrates that the “no collision” case is no longer valid when r=6.

Even though the distribution is only improved under certain conditions (user count less than 200), the proposed inner-code construction and detection algorithm, in conjunction with the outer error correction technique, can improve the overall performance. This will be discussed in more detail in the next subsection.

### 7.2. The Overall Performance of the SPC-Based CCS for URA System

#### 7.2.1. *t*-Tree Code as the Outer Code

**Figure 7:**
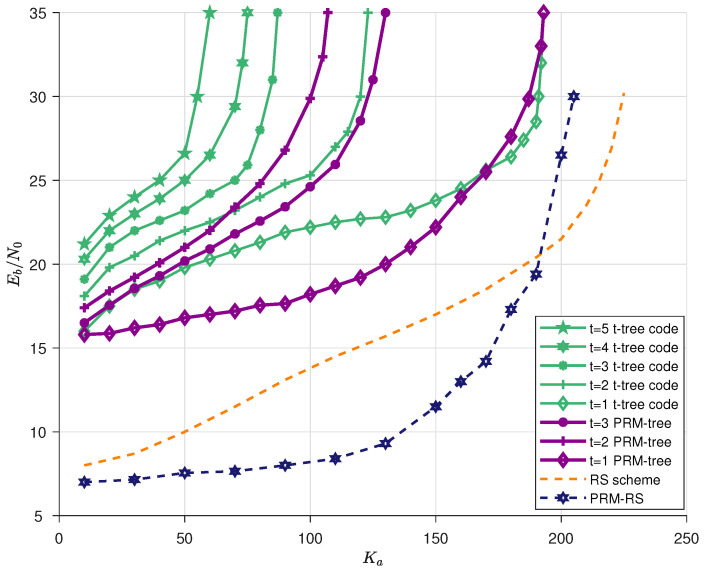
The performance for quasi-static Rayleigh fading channel is expressed as the required $E_{b}/N_{0}$ per active user vs. the number of active users $K_{a}$, given the channel uses T=32,768, the users’ messages B=10 bits and the target error probability $P_{e}=10^{-1}$, $P_{f}=10^{-3}$. The optimal parameters in our PRM-based scheme are carefully chosen to minimize the required $E_{b}/N_{0}$. The curves are presented as follows: *t*-tree code for t=1,⋯,5 from [22], PRM-tree scheme (optimal parameters are taken from the Table 1 and Table 2), Reed–Solomon scheme from [22] and PRM-RS scheme (optimal parameters are taken from Table 3).

To begin with, let us employ a decoder capable of correcting up to *t* errors as the outer code in different URA schemes, i.e., “*t*-tree code” and “PRM-tree”. The benchmarks called “*t*-tree code” from [22] are plotted by the green lines in Figure 7. We then evaluated the proposed “PRM-tree” scheme, i.e., the proposed PRM sequence is employed as the inner codeword, in which the former $x_{p}$-bit message is filled with the selected SPC, and the remaining *J*-bit is used to transport the user’s messages; moreover, the decoder capable of correcting *t* errors continues to make up the outer code. Following [22], we employ the greedy information bits allocation method, assigning the maximum number of information bits at each subsequent slot keeping the $E[|V_{l}\left|\right]\leq 2^{25}$ constraint to find the minimum $E_{b}/N_{0}$ (see Section VII in [22] for more details). Based on this, parameters of *H*, *N*, $N_{T}$, *m*, $\ddot{m}$, *G*, *J* and $x_{p}$ in the proposed URA scheme are also considered.

The PRM capacity formula under one rank Υ is shown below:

(50) |Γ|Υ=mΥ·2m·2Υ(Υ+1)2,

it is worth noting that ${\left|\Gamma \right|}_{\Upsilon }$ and ${\left|\Omega \right|}_{r}$ share the similar Formula (48).

Increasing *t* requires a longer outer-code length *H*, where $H=2^{r}$. By substituting $\ddot{m}$ and *r* into (48), it is easy to see that the *H* value directly controls the SPC capacity ${\left|\Omega \right|}_{r}$. On this basis, the optimal setups of *H* for t=1 and t=2,3 are set to be 32 and 64, respectively. However, t=4,5 cases are not valid in our URA setups since H=64 is no longer sufficient for t=4,5 and thus H=128 is the next choice (*H* must be a power of two). It can be seen from Equation (42) that the value of ${\left|\Omega \right|}_{r}$ related to H=128 requires a larger $\ddot{m}$ to preserve the average number of probable appearances in each slot, whereas $N=2^{m}$ would become smaller under the constraint of $T=N_{T}\cdot N$ (*T* is fixed and $N_{T}=2^{\ddot{m}}$), which results in the collapse of the inner-code performance. Table 1 presents the optimal *G*-ary, $x_{p}$ and *J*-ary for different *t*, as well as the outer-code length (and rate). Optimal parameters for the overall system are carefully selected as in Table 2. Simulations are shown in Figure 7, where purple lines illustrate the resulting energy efficiency for t=1,2 and 3 of “PRM-tree”. Based on the simulations, we can infer the following observations:

1. PRM sequence for a given rank suffices for a user’s message delivery, indicating that the PRM codebook is highly spectral efficient due to its large sequence space.
2. The outer-code length H=32(r=5) is used for t=1 and 64(r=6) for t=2,3. Substituting r=5,6 and $\ddot{m}=8$ into (38), we observe that the latter case (r=6) performs relatively poorly since it has a greater number of simultaneous appearances, which leads to inner-code failure at $K_{a}=200$ for t=1 and, for t=2,3, $K_{a}=150$. This result is consistent with Figure 6.
3. For the curves of t=2,3, the “PRM-tree” scheme corrects the case of the “*t*-tree code” scheme in which the overall performance degrades as *t* increases, i.e., an outer code with a larger *t* performs better on the condition that the inner code has the same length and the required path number is sufficient (“PRM-tree” schemes use $2^{25}$ paths and $2^{10}$ paths for “t-tree code”).

#### 7.2.2. PRM-Based Reed–Solomon Scheme

**Figure 8:**
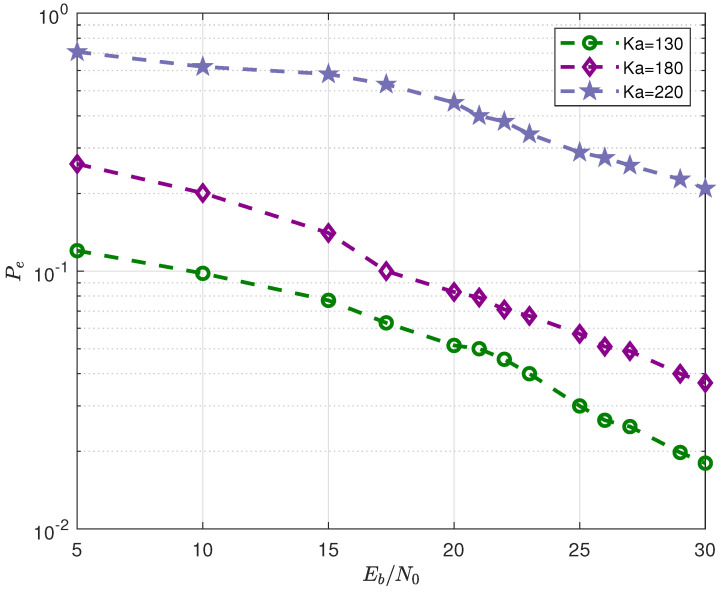
Probability of error $P_{e}$ vs. $E_{b}/N_{0}$ for three system loads (number of active users), $K_{a}=130,180$ and 220.

A “PRM-RS” scheme based on the inner PRM code in conjunction with an outer Reed–Solomon code (as illustrated in Algorithm 2) is simulated in this section, and the per-user error probability $P_{e}$ as a function of $E_{b}/N_{0}$ for $K_{a}=130,180$ and 200 for this “PRM-RS” scheme is obtained (in Figure 8). The optimal system parameters are depicted in Table 3. It can be seen from Figure 8 that:

1. The minimum $P_{e}$ exceeds 0.1 when the user count reaches 220.
2. The simulation curves in Figure 8 are consistent with the bar chart performances in Figure 6, i.e., when the number of users exceeds 200, the number of simultaneous transmissions exceeds the identifiable maximum.

The energy efficiency is the minimum energy required to serve $K_{a}$ users with PUPE less than 0.1, which is depicted in the blue line in Figure 7 under our optimal setups (see Table 3). We also need to adjust the number of CRC bits $b_{c}$ to suppress falsely detected messages following the same method as operated in [22]. The benchmark is the RS scheme in [22] which is plotted using an orange line.

The increase of *r* necessitates a lengthier $\ddot{m}$ to maintain the SPC pool capacity and the length of the inner code cannot be increased due to the channel uses *T* constraint. Furthermore, the decrease in inner-code length also weakens its performance. Therefore, we fix the SPC capacity as ${\left|\Omega \right|}_{r}$ with $\ddot{m}=8$ and r=7. On this basis, when the prefix length $x_{p}$ is fixed, a rise in value *G* leads to a larger *J*, which directly increases the outer code rate. Additionally, an increase in the outer code rate would weaken its performance. In light of this, carefully selecting the *G* value is critical. Simulation demonstrates that it is more efficient than the original RS when $K_{a}$ is less than 185, even with a constant inner-code length for different user counts.

## 8. Conclusions

In this paper, We addressed a general framework for CCS under the packetized and slotted transmission protocol based on inner PRM codes and outer error correction codes. First, we propose PRM sequences to transmit information chunks in slots since their geometry property facilitates computationally efficient PRM detection. Furthermore, we extend the space segmenting strategy of PRM sequences to construct a predefined dictionary of SPCs for slot occupation and the SPC selection mechanism is prescribed by an information segment for each user. The factors influencing slot-based PRM detection performance are discussed. We validate the error probability advantage of our proposed PRM-based URA scheme through simulation. We further proposed to use list recoverable codes correcting *t* errors in the coded compressed sensing scheme. Specifically, we propose two practical constructions of outer codes. The first one is a modification of the tree code. It utilizes the PRM as inner codes and adds a SPC, and the major difference is a decoder capable of correcting up to *t* errors. The second one is based on the Reed–Solomon codes and Guruswami–Sudan list decoding algorithm. The optimal setups are determined to minimize SNR by optimizing the inner and outer codes jointly. Both schemes provide better energy efficiency compared with the benchmark. In the following study, we will perform a matrix decomposition of the Clifford matrix $G_{F}$ related to the patterned Reed–Muller sequence and view this decomposition process as a reconstruction of the transmitter design. Moreover, the model is extended to accommodate multiple antennas for the URA system. We will also consider an iterative decoding algorithm to solve the signal recovery at the receiver.

## Figures and Tables

**Table 1:** Optimal *G*-ary, $x_{p}$ and *J*-ary, as well as the outer-code length *H* (and rate $R=\frac{B}{H\log_{2}J}$) for different *t*.

*t*	*G*	$x_{p}$	*J*	$K_{a}=50$	$K_{a}=100$	$K_{a}=150$	$K_{a}=200$
t=1	19	9	10	32 (0.3125)	32 (0.3125)	32 (0.2232)	32 (0.3125)
t=2	15	7	8	64 (0.1953)	64 (0.1953)	−	−
t=3	15	7	8	64 (0.1953)	64 (0.1953)	−	−

**Table 2:** URA system parameters for “PRM-tree” scheme.

Parameter Description	Specific Value
Transmit a message of size, *B*	100 bits
PRM sequence length, $N=2^{m}$	$2^{7}\left(m=7\right)$
The length of RS codes, $H=2^{r}$	$2^{5}\left(r=5\right)/2^{6}\left(r=6\right)$
The number of slots, $N_{T}$	$2^{8}\left(\ddot{m}=8\right)$
The number of complex channel uses, *T*	$2^{15}$
The capacity of slot-occupation pool, ${\left|\Omega \right|}_{r}$	448 (r=5)/112 (r=6)
The length of slot-occupation control, $x_{p}$	9/7
*G*-ary	19/15
*J*-ary	10/8
The capacity of PRM codebook, $|\Gamma_{\Upsilon }|$	286,720 (Υ=3)/21,504 (Υ=2)
The code rate	0.3125/0.1953

**Table 3:** URA system parameters for “PRM-RS” scheme.

Parameter Description	Specific Value
Transmit a message of size, *B*	100 bits
PRM sequence length, $N=2^{m}$	$2^{7}\left(m=7\right)$
The length of RS codes, $H=2^{r}$	$2^{5}\left(r=5\right)$
The number of slots, $N_{T}$	$2^{8}\left(\ddot{m}=8\right)$
The number of complex channel uses, *T*	$2^{15}$
The capacity of slot-occupation pool, ${\left|\Omega \right|}_{r}$	448 (r=5)
The length of slot-occupation control, $x_{p}$	9
*G*-ary	15
*J*-ary	6
The capacity of PRM codebook, $|\Gamma_{\Upsilon }|$	21,504 (Υ=2)
The code rate of RS	0.5208

## Data Availability

Not applicable.
